# Triphen­yl(pyrrolidine-1-carbodi­thio­ato-κ*S*)tin(IV)

**DOI:** 10.1107/S1600536813022472

**Published:** 2013-08-17

**Authors:** Muhammad Sirajuddin, Noor Uddin, Saqib Ali

**Affiliations:** a Department of Chemistry, Quaid-i-Azam University, Islamabad, Pakistan

## Abstract

In the title compound, [Sn(C_6_H_5_)_3_(C_5_H_8_NS_2_)], the Sn^IV^ atom adopts a distorted SnC_3_S tetra­hedral coordination geometry [spread of bond angles = 94.43 (7)–120.74 (7)°]. A short intra­molecular Sn⋯S contact [3.0270 (9) Å] occurs and two intra­molecular C—H⋯S inter­actions help to establish the conformation. Three of the methyl­ene groups of the pyrrolidine-1-carbodi­thio­ate ligand are disordered over two sets of sites of equal occupancy. In the crystal, very weak C—H⋯S inter­actions link the mol­ecules into a three-dimensional network, with both S atoms acting as acceptors.

## Related literature

For background to the structures and applications of organotin compounds, see: Abbas *et al.* (2013 ▶); Pellerito & Nagy (2002 ▶); Ronconi *et al.* (2005 ▶); Shahzadi *et al.* (2006 ▶, 2008 ▶); Sirajuddin *et al.* (2012 ▶).
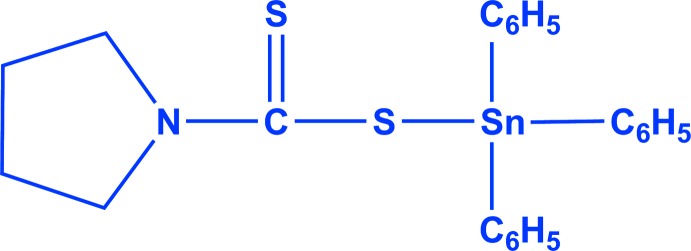


## Experimental

### 

#### Crystal data

[Sn(C_6_H_5_)_3_(C_5_H_8_NS_2_)]
*M* *_r_* = 496.23
Monoclinic, 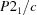

*a* = 12.3467 (4) Å
*b* = 10.3227 (3) Å
*c* = 17.0611 (6) Å
β = 90.864 (2)°
*V* = 2174.21 (12) Å^3^
*Z* = 4
Mo *K*α radiation
μ = 1.38 mm^−1^
*T* = 296 K
0.32 × 0.26 × 0.24 mm

#### Data collection

Bruker Kappa APEXII CCD diffractometer
Absorption correction: multi-scan (*SADABS*; Bruker, 2005 ▶) *T*_min_ = 0.667, *T*_max_ = 0.734
16466 measured reflections
4265 independent reflections
3567 reflections with *I* > 2σ(*I*)
*R*_int_ = 0.020

#### Refinement

*R*[*F* ^2^ > 2σ(*F* ^2^)] = 0.022
*wR*(*F* ^2^) = 0.062
*S* = 1.01
4265 reflections
253 parameters
3 restraints
H-atom parameters constrained
Δρ_max_ = 0.39 e Å^−3^
Δρ_min_ = −0.47 e Å^−3^

### 

Data collection: *APEX2* (Bruker, 2007 ▶); cell refinement: *SAINT* (Bruker, 2007 ▶); data reduction: *SAINT*; program(s) used to solve structure: *SHELXS97* (Sheldrick, 2008 ▶); program(s) used to refine structure: *SHELXL2012* (Sheldrick, 2012 ▶); molecular graphics: *ORTEP-3 for Windows* (Farrugia, 2012 ▶) and *PLATON* (Spek, 2009 ▶); software used to prepare material for publication: *WinGX* (Farrugia, 2012 ▶) and *PLATON*.

## Supplementary Material

Crystal structure: contains datablock(s) I, New_Global_Publ_Block. DOI: 10.1107/S1600536813022472/hb7122sup1.cif

Structure factors: contains datablock(s) I. DOI: 10.1107/S1600536813022472/hb7122Isup2.hkl

Supplementary material file. DOI: 10.1107/S1600536813022472/hb7122Isup3.cml

Additional supplementary materials:  crystallographic information; 3D view; checkCIF report

## Figures and Tables

**Table 1:** Selected bond lengths (Å)

Sn1—C6	2.160 (2)
Sn1—C12	2.134 (2)
Sn1—C18	2.149 (2)
Sn1—S1	2.4710 (7)

**Table 2:** Hydrogen-bond geometry (Å, °)

*D*—H⋯*A*	*D*—H	H⋯*A*	*D*⋯*A*	*D*—H⋯*A*
C7—H7⋯S1	0.93	2.75	3.384 (3)	127
C23—H23⋯S2	0.93	2.77	3.424 (3)	129
C2—H2*B*⋯S2^i^	0.97	2.96	3.759 (3)	141
C5—H5*B*⋯S1^ii^	0.97	2.98	3.750 (3)	137

Symmetry codes: (i) 
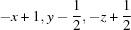
; (ii) 
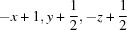
.

**Fig. 1.**
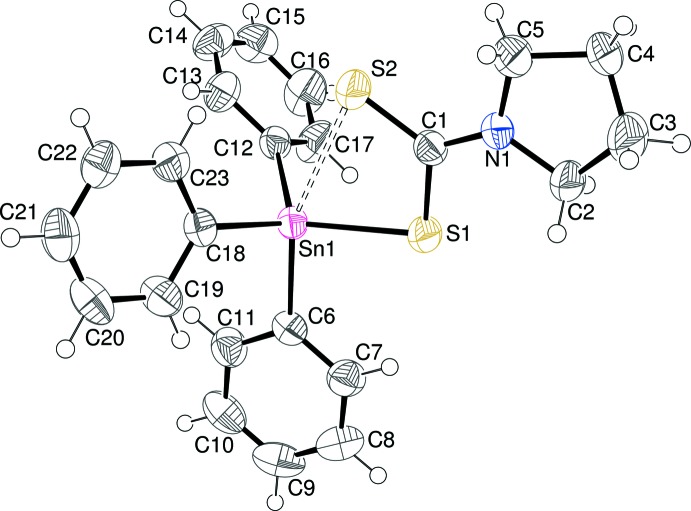
View of the title compound with displacement ellipsoids drawn at the 50% probability level.

**Fig. 2.**
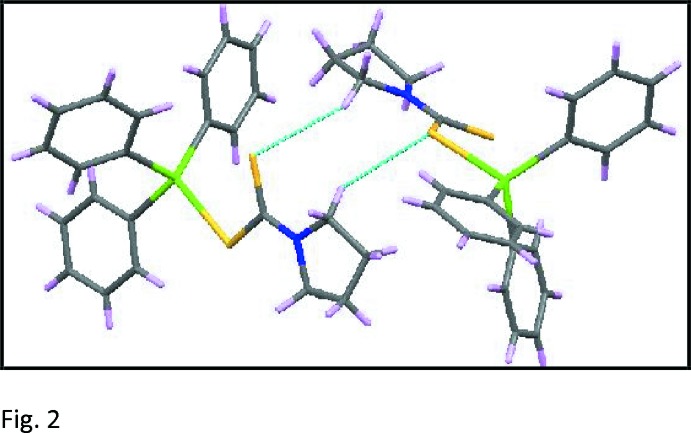
The partial packing, which shows that molecules form dimers due to C—H···S bonds.

## supplementary crystallographic information

### 1. Experimental

Stoichiometric amount of sodium salt of pyrrolidine-1-carbodithioate was suspended in dry toluene and to it calculated amount of triphenyltin(IV) chloride, Ph_3_SnCl, (5 mmol of each, 1:1 ratio) was added. The mixture was stirred and refluxed for 3–4 h, and then it was cooled and filtered to remove NaCl. The filterate was rotary evaporated to get the product which was recystallized from chloroform solution to yield colourless prisms.

### 2. Refinement

The H-atoms were positioned geometrically (C–H = 0.93–0.96 Å) and refined as riding with *U*_iso_(H) = *xU*_eq_(C), where *x* = 1.5 for methyl and *x* = 1.2 for other H-atoms.

### Crystal data

**Table:**

[Sn(C_6_H_5_)_3_(C_5_H_8_NS_2_)]	*Z* = 4
*M**_r_* = 496.23	*F*(000) = 1000
Monoclinic, *P*2_1_/*c*	*D*_x_ = 1.516 Mg m^−^^3^
*a* = 12.3467 (4) Å	Mo *K*α radiation, λ = 0.71073 Å
*b* = 10.3227 (3) Å	µ = 1.38 mm^−^^1^
*c* = 17.0611 (6) Å	*T* = 296 K
β = 90.864 (2)°	Prism, colourless
*V* = 2174.21 (12) Å^3^	0.32 × 0.26 × 0.24 mm

### Data collection

**Table:**

Bruker Kappa APEXII CCD diffractometer	3567 reflections with *I* > 2σ(*I*)
Radiation source: fine-focus sealed tube	*R*_int_ = 0.020
ω scans	θ_max_ = 26.0°, θ_min_ = 1.7°
Absorption correction: multi-scan (*SADABS*; Bruker, 2005)	*h* = −15→12
*T*_min_ = 0.667, *T*_max_ = 0.734	*k* = −12→12
16466 measured reflections	*l* = −20→21
4265 independent reflections	

### Refinement

**Table:**

Refinement on *F*^2^	3 restraints
Least-squares matrix: full	Hydrogen site location: inferred from neighbouring sites
*R*[*F*^2^ > 2σ(*F*^2^)] = 0.022	H-atom parameters constrained
*wR*(*F*^2^) = 0.062	*w* = 1/[σ^2^(*F*_o_^2^) + (0.0255*P*)^2^ + 1.8846*P*] where *P* = (*F*_o_^2^ + 2*F*_c_^2^)/3
*S* = 1.01	(Δ/σ)_max_ = 0.001
4265 reflections	Δρ_max_ = 0.39 e Å^−^^3^
253 parameters	Δρ_min_ = −0.47 e Å^−^^3^

### Special details

**Table:**

Geometry. All e.s.d.'s (except the e.s.d. in the dihedral angle between two l.s. planes) are estimated using the full covariance matrix. The cell e.s.d.'s are taken into account individually in the estimation of e.s.d.'s in distances, angles and torsion angles; correlations between e.s.d.'s in cell parameters are only used when they are defined by crystal symmetry. An approximate (isotropic) treatment of cell e.s.d.'s is used for estimating e.s.d.'s involving l.s. planes.

### Fractional atomic coordinates and isotropic or equivalent isotropic displacement parameters (Å^2^)

**Table:**

	*x*	*y*	*z*	*U*_iso_*/*U*_eq_	Occ. (<1)
C1	0.43914 (19)	0.5449 (2)	0.19935 (15)	0.0395 (5)	
C2	0.6278 (2)	0.4746 (3)	0.22777 (17)	0.0523 (7)	
H2A	0.6637	0.5121	0.1830	0.063*	
H2B	0.6145	0.3835	0.2178	0.063*	
C3	0.6941 (3)	0.4935 (4)	0.3019 (2)	0.0806 (11)	0.5
H3A	0.6860	0.4194	0.3363	0.097*	0.5
H3B	0.7701	0.5026	0.2896	0.097*	0.5
C4	0.6549 (5)	0.6099 (7)	0.3397 (4)	0.0552 (17)	0.5
H4A	0.6913	0.6861	0.3198	0.066*	0.5
H4B	0.6665	0.6055	0.3960	0.066*	0.5
C5	0.5338 (2)	0.6133 (3)	0.31911 (18)	0.0670 (9)	0.5
H5A	0.4918	0.5703	0.3591	0.080*	0.5
H5B	0.5083	0.7017	0.3134	0.080*	0.5
C3'	0.6941 (3)	0.4935 (4)	0.3019 (2)	0.0806 (11)	0.5
H3'1	0.7241	0.4113	0.3192	0.097*	0.5
H3'2	0.7536	0.5525	0.2923	0.097*	0.5
C4'	0.6248 (7)	0.5461 (10)	0.3617 (4)	0.083 (3)	0.5
H4'1	0.6646	0.6069	0.3945	0.100*	0.5
H4'2	0.5970	0.4773	0.3945	0.100*	0.5
C5'	0.5338 (2)	0.6133 (3)	0.31911 (18)	0.0670 (9)	0.5
H5'1	0.4668	0.6064	0.3478	0.080*	0.5
H5'2	0.5503	0.7041	0.3109	0.080*	0.5
C6	0.2627 (2)	0.3725 (2)	−0.03124 (14)	0.0419 (5)	
C7	0.3521 (2)	0.2959 (3)	−0.04850 (16)	0.0520 (6)	
H7	0.4133	0.2995	−0.0162	0.062*	
C8	0.3512 (3)	0.2147 (3)	−0.11275 (18)	0.0650 (8)	
H8	0.4119	0.1648	−0.1234	0.078*	
C9	0.2618 (3)	0.2069 (3)	−0.16087 (18)	0.0692 (9)	
H9	0.2611	0.1504	−0.2033	0.083*	
C10	0.1735 (3)	0.2826 (3)	−0.14625 (18)	0.0677 (9)	
H10	0.1131	0.2787	−0.1794	0.081*	
C11	0.1740 (2)	0.3653 (3)	−0.08204 (16)	0.0545 (7)	
H11	0.1137	0.4169	−0.0729	0.065*	
C12	0.22278 (19)	0.6899 (2)	0.03333 (14)	0.0402 (5)	
C13	0.1372 (2)	0.7587 (3)	0.06383 (16)	0.0508 (6)	
H13	0.0922	0.7191	0.0999	0.061*	
C14	0.1174 (2)	0.8859 (3)	0.04166 (17)	0.0583 (7)	
H14	0.0593	0.9305	0.0627	0.070*	
C15	0.1829 (3)	0.9458 (3)	−0.01099 (19)	0.0636 (8)	
H15	0.1707	1.0316	−0.0250	0.076*	
C16	0.2661 (3)	0.8784 (3)	−0.0427 (2)	0.0751 (10)	
H16	0.3101	0.9182	−0.0793	0.090*	
C17	0.2860 (2)	0.7515 (3)	−0.02123 (19)	0.0641 (8)	
H17	0.3430	0.7069	−0.0439	0.077*	
C18	0.12332 (19)	0.4161 (2)	0.13624 (14)	0.0404 (5)	
C19	0.0755 (2)	0.3023 (3)	0.11048 (17)	0.0552 (7)	
H19	0.1057	0.2566	0.0692	0.066*	
C20	−0.0173 (3)	0.2559 (3)	0.1459 (2)	0.0666 (8)	
H20	−0.0485	0.1791	0.1282	0.080*	
C21	−0.0632 (2)	0.3217 (3)	0.20636 (18)	0.0618 (8)	
H21	−0.1261	0.2908	0.2291	0.074*	
C22	−0.0157 (2)	0.4337 (3)	0.23320 (18)	0.0579 (7)	
H22	−0.0459	0.4783	0.2749	0.069*	
C23	0.0769 (2)	0.4804 (3)	0.19850 (17)	0.0490 (6)	
H23	0.1085	0.5562	0.2173	0.059*	
N1	0.52626 (16)	0.5437 (2)	0.24462 (13)	0.0433 (5)	
S1	0.44472 (5)	0.45725 (7)	0.11166 (4)	0.04934 (16)	
S2	0.32513 (6)	0.62405 (8)	0.22271 (5)	0.0592 (2)	
Sn1	0.25469 (2)	0.49609 (2)	0.07066 (2)	0.03669 (7)	

### Atomic displacement parameters (Å^2^)

**Table:**

	*U*^11^	*U*^22^	*U*^33^	*U*^12^	*U*^13^	*U*^23^
C1	0.0373 (12)	0.0355 (12)	0.0456 (14)	−0.0018 (10)	−0.0042 (10)	0.0014 (10)
C2	0.0416 (14)	0.0586 (16)	0.0563 (17)	0.0107 (12)	−0.0099 (12)	−0.0057 (13)
C3	0.059 (2)	0.105 (3)	0.077 (2)	0.0268 (19)	−0.0302 (18)	−0.022 (2)
C4	0.045 (4)	0.067 (4)	0.053 (4)	0.002 (3)	−0.018 (3)	−0.010 (3)
C5	0.0516 (17)	0.086 (2)	0.0628 (19)	0.0106 (16)	−0.0160 (14)	−0.0287 (17)
C3'	0.059 (2)	0.105 (3)	0.077 (2)	0.0268 (19)	−0.0302 (18)	−0.022 (2)
C4'	0.065 (5)	0.130 (8)	0.053 (5)	0.010 (5)	−0.023 (4)	−0.007 (5)
C5'	0.0516 (17)	0.086 (2)	0.0628 (19)	0.0106 (16)	−0.0160 (14)	−0.0287 (17)
C6	0.0453 (13)	0.0420 (13)	0.0383 (13)	−0.0054 (11)	−0.0002 (10)	0.0002 (10)
C7	0.0550 (16)	0.0483 (15)	0.0527 (16)	0.0029 (13)	−0.0052 (13)	−0.0009 (13)
C8	0.083 (2)	0.0484 (16)	0.0636 (19)	0.0115 (15)	0.0071 (17)	−0.0067 (14)
C9	0.107 (3)	0.0500 (17)	0.0508 (17)	−0.0113 (18)	−0.0021 (18)	−0.0096 (14)
C10	0.075 (2)	0.074 (2)	0.0537 (18)	−0.0202 (18)	−0.0182 (16)	−0.0070 (16)
C11	0.0488 (15)	0.0648 (18)	0.0496 (16)	−0.0037 (13)	−0.0036 (12)	−0.0038 (13)
C12	0.0369 (12)	0.0403 (13)	0.0433 (13)	0.0008 (10)	−0.0051 (10)	0.0001 (10)
C13	0.0476 (14)	0.0576 (16)	0.0471 (15)	0.0093 (12)	0.0033 (12)	0.0084 (12)
C14	0.0624 (18)	0.0587 (17)	0.0534 (17)	0.0233 (14)	−0.0073 (14)	−0.0023 (14)
C15	0.077 (2)	0.0455 (15)	0.068 (2)	0.0063 (15)	−0.0095 (17)	0.0103 (15)
C16	0.071 (2)	0.061 (2)	0.094 (3)	−0.0021 (17)	0.0201 (19)	0.0274 (18)
C17	0.0563 (17)	0.0556 (17)	0.081 (2)	0.0090 (14)	0.0231 (16)	0.0119 (15)
C18	0.0362 (12)	0.0435 (13)	0.0413 (13)	−0.0033 (10)	−0.0030 (10)	0.0049 (11)
C19	0.0589 (17)	0.0559 (16)	0.0507 (16)	−0.0126 (14)	0.0020 (13)	−0.0050 (13)
C20	0.0636 (19)	0.0608 (18)	0.075 (2)	−0.0271 (16)	−0.0033 (16)	0.0026 (16)
C21	0.0462 (15)	0.073 (2)	0.0663 (19)	−0.0117 (15)	0.0057 (14)	0.0158 (16)
C22	0.0481 (16)	0.0658 (19)	0.0601 (18)	0.0026 (14)	0.0113 (13)	0.0057 (15)
C23	0.0455 (14)	0.0476 (15)	0.0540 (16)	−0.0032 (11)	0.0033 (12)	0.0001 (12)
N1	0.0353 (11)	0.0441 (11)	0.0502 (12)	0.0024 (9)	−0.0086 (9)	−0.0053 (10)
S1	0.0406 (3)	0.0563 (4)	0.0507 (4)	0.0078 (3)	−0.0098 (3)	−0.0116 (3)
S2	0.0398 (3)	0.0698 (5)	0.0678 (5)	0.0120 (3)	−0.0098 (3)	−0.0196 (4)
Sn1	0.03201 (10)	0.03739 (10)	0.04059 (10)	−0.00054 (6)	−0.00174 (7)	−0.00022 (7)

### Geometric parameters (Å, º)

**Table:**

C1—N1	1.315 (3)	C11—H11	0.9300
C1—S2	1.681 (2)	C12—C17	1.379 (4)
C1—S1	1.750 (3)	C12—C13	1.381 (3)
C2—N1	1.474 (3)	C13—C14	1.387 (4)
C2—C3	1.509 (4)	C13—H13	0.9300
C2—H2A	0.9700	C14—C15	1.365 (4)
C2—H2B	0.9700	C14—H14	0.9300
C3—C4	1.451 (7)	C15—C16	1.360 (5)
C3—H3A	0.9700	C15—H15	0.9300
C3—H3B	0.9700	C16—C17	1.381 (4)
C4—C5	1.532 (7)	C16—H16	0.9300
C4—H4A	0.9700	C17—H17	0.9300
C4—H4B	0.9700	C18—C23	1.384 (4)
C5—N1	1.462 (3)	C18—C19	1.384 (4)
C5—H5A	0.9700	C19—C20	1.388 (4)
C5—H5B	0.9700	C19—H19	0.9300
C4'—H4'1	0.9700	C20—C21	1.366 (4)
C4'—H4'2	0.9700	C20—H20	0.9300
C6—C11	1.389 (4)	C21—C22	1.372 (4)
C6—C7	1.393 (4)	C21—H21	0.9300
C7—C8	1.380 (4)	C22—C23	1.382 (4)
C7—H7	0.9300	C22—H22	0.9300
C8—C9	1.368 (5)	C23—H23	0.9300
C8—H8	0.9300	Sn1—C6	2.160 (2)
C9—C10	1.367 (5)	Sn1—C12	2.134 (2)
C9—H9	0.9300	Sn1—C18	2.149 (2)
C10—C11	1.389 (4)	Sn1—S1	2.4710 (7)
C10—H10	0.9300		
			
N1—C1—S2	123.1 (2)	C17—C12—C13	117.5 (2)
N1—C1—S1	117.06 (18)	C17—C12—Sn1	121.97 (19)
S2—C1—S1	119.85 (14)	C13—C12—Sn1	120.56 (19)
N1—C2—C3	103.1 (2)	C12—C13—C14	121.1 (3)
N1—C2—H2A	111.1	C12—C13—H13	119.4
C3—C2—H2A	111.1	C14—C13—H13	119.4
N1—C2—H2B	111.1	C15—C14—C13	120.3 (3)
C3—C2—H2B	111.1	C15—C14—H14	119.9
H2A—C2—H2B	109.1	C13—C14—H14	119.9
C4—C3—C2	107.4 (4)	C16—C15—C14	119.3 (3)
C4—C3—H3A	110.2	C16—C15—H15	120.4
C2—C3—H3A	110.2	C14—C15—H15	120.4
C4—C3—H3B	110.2	C15—C16—C17	120.8 (3)
C2—C3—H3B	110.2	C15—C16—H16	119.6
H3A—C3—H3B	108.5	C17—C16—H16	119.6
C3—C4—C5	104.3 (4)	C12—C17—C16	121.0 (3)
C3—C4—H4A	110.9	C12—C17—H17	119.5
C5—C4—H4A	110.9	C16—C17—H17	119.5
C3—C4—H4B	110.9	C23—C18—C19	118.1 (2)
C5—C4—H4B	110.9	C23—C18—Sn1	122.71 (18)
H4A—C4—H4B	108.9	C19—C18—Sn1	118.86 (19)
N1—C5—C4	103.7 (3)	C18—C19—C20	120.4 (3)
N1—C5—H5A	111.0	C18—C19—H19	119.8
C4—C5—H5A	111.0	C20—C19—H19	119.8
N1—C5—H5B	111.0	C21—C20—C19	120.8 (3)
C4—C5—H5B	111.0	C21—C20—H20	119.6
H5A—C5—H5B	109.0	C19—C20—H20	119.6
H4'1—C4'—H4'2	108.7	C20—C21—C22	119.4 (3)
C11—C6—C7	117.2 (2)	C20—C21—H21	120.3
C11—C6—Sn1	119.27 (19)	C22—C21—H21	120.3
C7—C6—Sn1	123.48 (19)	C21—C22—C23	120.2 (3)
C8—C7—C6	121.1 (3)	C21—C22—H22	119.9
C8—C7—H7	119.5	C23—C22—H22	119.9
C6—C7—H7	119.5	C18—C23—C22	121.1 (3)
C9—C8—C7	120.6 (3)	C18—C23—H23	119.4
C9—C8—H8	119.7	C22—C23—H23	119.4
C7—C8—H8	119.7	C1—N1—C5	123.2 (2)
C10—C9—C8	119.7 (3)	C1—N1—C2	125.5 (2)
C10—C9—H9	120.2	C5—N1—C2	111.4 (2)
C8—C9—H9	120.2	C1—S1—Sn1	96.24 (8)
C9—C10—C11	120.1 (3)	C12—Sn1—C18	112.21 (9)
C9—C10—H10	120.0	C12—Sn1—C6	108.90 (9)
C11—C10—H10	120.0	C18—Sn1—C6	103.66 (9)
C6—C11—C10	121.3 (3)	C12—Sn1—S1	114.00 (6)
C6—C11—H11	119.4	C18—Sn1—S1	120.74 (7)
C10—C11—H11	119.4	C6—Sn1—S1	94.43 (7)
			
N1—C2—C3—C4	−24.6 (5)	C23—C18—C19—C20	0.9 (4)
C2—C3—C4—C5	32.6 (6)	Sn1—C18—C19—C20	−172.5 (2)
C3—C4—C5—N1	−27.4 (6)	C18—C19—C20—C21	0.2 (5)
C11—C6—C7—C8	−1.1 (4)	C19—C20—C21—C22	−1.2 (5)
Sn1—C6—C7—C8	177.4 (2)	C20—C21—C22—C23	1.0 (5)
C6—C7—C8—C9	−0.5 (5)	C19—C18—C23—C22	−1.1 (4)
C7—C8—C9—C10	1.6 (5)	Sn1—C18—C23—C22	172.0 (2)
C8—C9—C10—C11	−1.1 (5)	C21—C22—C23—C18	0.2 (4)
C7—C6—C11—C10	1.6 (4)	S2—C1—N1—C5	−1.3 (4)
Sn1—C6—C11—C10	−176.9 (2)	S1—C1—N1—C5	179.4 (2)
C9—C10—C11—C6	−0.5 (5)	S2—C1—N1—C2	179.1 (2)
C17—C12—C13—C14	−1.4 (4)	S1—C1—N1—C2	−0.2 (4)
Sn1—C12—C13—C14	178.2 (2)	C4—C5—N1—C1	−167.1 (4)
C12—C13—C14—C15	−0.2 (4)	C4—C5—N1—C2	12.6 (4)
C13—C14—C15—C16	1.5 (5)	C3—C2—N1—C1	−173.9 (3)
C14—C15—C16—C17	−1.2 (6)	C3—C2—N1—C5	6.5 (3)
C13—C12—C17—C16	1.8 (5)	N1—C1—S1—Sn1	176.18 (19)
Sn1—C12—C17—C16	−177.9 (3)	S2—C1—S1—Sn1	−3.10 (16)
C15—C16—C17—C12	−0.5 (6)		

### Hydrogen-bond geometry (Å, º)

**Table:**

*D*—H···*A*	*D*—H	H···*A*	*D*···*A*	*D*—H···*A*
C7—H7···S1	0.93	2.75	3.384 (3)	127
C23—H23···S2	0.93	2.77	3.424 (3)	129
C2—H2*B*···S2^i^	0.97	2.96	3.759 (3)	141
C5—H5*B*···S1^ii^	0.97	2.98	3.750 (3)	137

Symmetry codes: (i) −*x*+1, *y*−1/2, −*z*+1/2; (ii) −*x*+1, *y*+1/2, −*z*+1/2.
